# Prevalence and Characterization of Self-Reported Gluten Sensitivity in The Netherlands

**DOI:** 10.3390/nu8110714

**Published:** 2016-11-08

**Authors:** Tom van Gils, Petula Nijeboer, Catharina E. IJssennagger, David S. Sanders, Chris J. J. Mulder, Gerd Bouma

**Affiliations:** 1 Celiac Center Amsterdam, Department Gastroenterology and Hepatology, VU University Medical Center, P.O. Box 7057, 1007 MB Amsterdam, The Netherlands; p.nijeboer@vumc.nl (P.N.); c.ijssennagger@vumc.nl (C.E.I.); cjmulder@vumc.nl (C.J.J.M.); g.bouma@vumc.nl (G.B.); 2 Academic Unit of Gastroenterology, Royal Hallamshire Hospital, Sheffield S10 2JF, UK; David.Sanders@sth.nhs.uk

**Keywords:** gluten, non-celiac gluten sensitivity, non-celiac wheat sensitivity, celiac disease, irritable bowel syndrome, FODMAPs

## Abstract

Background: A growing number of individuals reports symptoms related to the ingestion of gluten-containing food in the absence of celiac disease. Yet the actual prevalence is not well established. Methods: Between April 2015 and March 2016, unselected adults visiting marketplaces, dental practices and a university in The Netherlands were asked to complete a modified validated questionnaire for self-reported gluten sensitivity (srGS). Results: Among the 785 adults enquired, two had celiac disease. Forty-nine (6.2%) reported symptoms related to the ingestion of gluten-containing food. These individuals were younger, predominantly female and lived more frequently in urban regions compared with the other respondents. Symptoms reported included bloating (74%), abdominal discomfort (49%) and flatulence (47%). A total of 23 (47%) srGS individuals reported having had tried a gluten-free or gluten-restricted diet. Abdominal discomfort related to fermentable oligosaccharide, disaccharide, monosaccharide and polyol (FODMAP)-containing food was more often reported in srGS individuals compared with the other respondents (73.5% vs. 21.7%, *p <* 0.001). Conclusion: Self-reported GS is common in The Netherlands, especially in younger individuals, females and urban regions, although the prevalence was lower than in a comparable recent UK study. It cannot be excluded that FODMAPs are in part responsible for these symptoms.

## 1. Introduction

The concept of a causal relationship between the ingestion of gluten and the occurrence of symptoms in the absence of celiac disease (CD) and wheat allergy was first described in the late 1970s by Cooper and Ellis [1,2]. This clinical entity has been termed non-celiac gluten or wheat sensitivity (NCGS or NCWS) [3,4]. Over the past several years, NCWS has gained significant interest and the number of individuals embracing a gluten-free diet is rapidly growing [5]. The discussion of whether or not gluten can cause symptoms in the absence of CD is confused by a popular phenomenon of people who avoid gluten-containing food in the light of a healthier lifestyle which is related to the fast growth of the gluten-free market [6]. This theory that grains by means of their composition are unhealthy, should be distinguished from the question as to whether gluten can cause symptoms in the absence of CD and wheat allergy.

As defined by the 2015 Salerno Expert’s Criteria [4], NCWS includes both intestinal and extra-intestinal symptoms which are related to the ingestion of gluten-containing food after exclusion of CD and wheat allergy. Most common symptoms include bloating, abdominal pain, diarrhea, tiredness and headache [4]. These symptoms display a significant overlap with the irritable bowel syndrome (IBS), which is one of the most common disorders in daily practice [7,8]. Although NCWS patients report that their symptoms are induced by gluten, it has been hypothesized that their symptoms may in fact be induced by other compounds in grains, among which a group of carbohydrates, referred to as fermentable oligo-, di-, monosaccharides and polyols (FODMAPs) have gained substantial interest [9]. 

Despite the overwhelming current interest in NCWS, the actual prevalence is difficult to establish in the absence of a gold standard. The number of studies that have addressed this question are sparse and the outcome varied widely between 0.6% and 13% [3,10,11,12,13,14,15,16]. 

Here, we studied the population prevalence of self-reported gluten sensitivity (srGS) in a large cohort of adults in the Dutch population. 

## 2. Materials and Methods

### 2.1. Participants

Between April 2015 and March 2016, individuals visiting markets, dental practices and a university in The Netherlands were asked to participate in a survey from the Gastroenterology and Hepatology department of the VU University Medical Center, Amsterdam (The Netherlands). There was no referral to the subject of the study during recruiting respondents. Participants completed a modified version of a previously validated questionnaire as described elsewhere [12]. Participation was anonymous and informed consent was given by completing the survey. Exclusion criteria included individuals under 18 years of age. At all sites a trained person was available to support the respondents when necessary. 

### 2.2. Questionnaire

**Figure 1:**
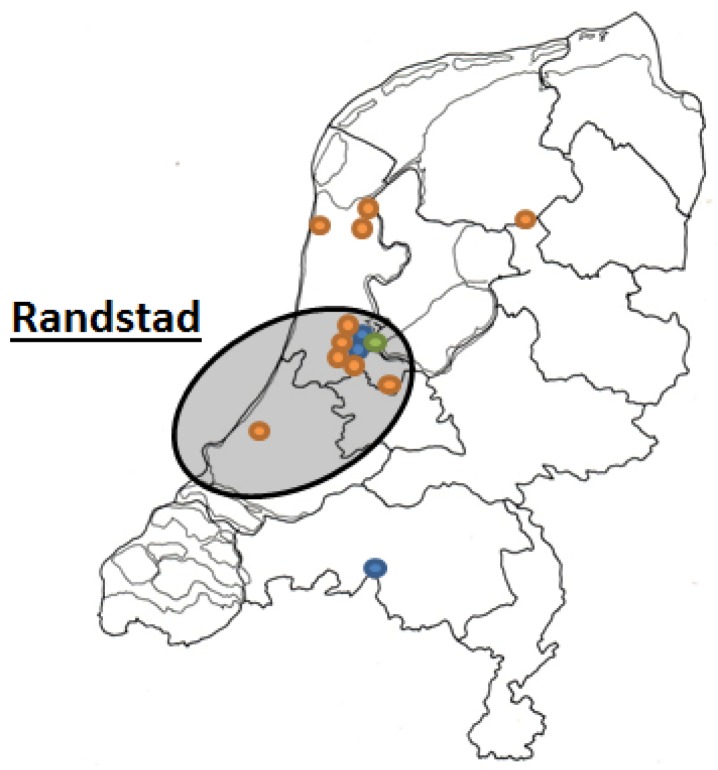
Sites where participants were recruited in The Netherlands. Orange: dental practice. Blue: market. Green: university. Circled: *Randstad* (considered as urban region).

The questionnaire was divided in four sections. The first comprised basic demographic information, including age, sex and education level. Living area was divided in urban and rural region based on the location where the respondents completed the questionnaire. Urban region was defined as the *Randstad*, a megalopolis in The Netherlands consisting of the four largest Dutch cities (Amsterdam, Rotterdam, The Hague and Utrecht). Rural area was defined as the area outside the *Randstad* and all these sites included small to middle-large villages with less than 20,000 inhabitants (Figure 1). A high education level was defined as having completed at least a higher professional education level or university education level. 

The second section screened for symptoms consistent with irritable bowel syndrome (IBS) in accordance with the Rome III criteria: recurrent abdominal pain or discomfort during the last six months. These should be present on three or more days a month together with two or three of the following situations: improvement with defecation, onset associated with a change in frequency of stool and/or onset associated with a change in shape of stool [7]. Participants were also asked about their medical history. 

The third section of the survey enquired about srGS and recognized related symptoms to gluten, as demonstrated by previous studies and those of expert opinions. In this section we also asked about the use of a GFD and other grain products in srGS as well as healthcare visits due to gluten-related symptoms. Respondents who did not reported gluten sensitivity or CD were considered as controls. 

The fourth and final section of the survey enquired about abdominal discomfort due to 17 different high FODMAP-containing supplements.

### 2.3. Statistical Analysis

Statistical analysis was performed using SPSS version 22.0 software (SPSS Inc., Chicago, IL, USA). Categorical variables were summarized by descriptive statistics, including total numbers, percentages and odds ratio (OR). Significance was analyzed using the two-tailed Fisher exact test. Continuous variables were summarized by mean and standard deviation (sd), with significant differences between two groups analyzed using the Independent Samples *T*-test. A *p*-value of less than 0.05 was considered as statistically significant. 

## 3. Results 

A total of 785 adults completed the questionnaire, of whom 66% were recruited in dental practices, 28% at markets and 6% at a university. Two individuals had an established diagnosis of CD and were excluded from further analysis. Mean age at the time of survey was 47 years old (sd: 18 years, range 18–90) with a slight predominance of women (60%). The majority of questionnaires (57%) was completed in the urban region. 

A total of 49 individuals (6.2%) indicated symptoms after the ingestion of gluten-containing foods. Such srGS individuals were younger (39 vs. 47 years old, *p =* 0.001), predominantly female (80% vs. 58%, *p <* 0.01) and mostly lived in the urban region (76% vs. 56%, *p <* 0.01) compared with the controls. Although not statistically significant, there was a trend for a higher education level in srGS individuals (49% with a high education level vs. 39%, not statistically significant). 

### 3.1. Characteristics of Self-Reported Gluten Sensitivity 

**Figure 2:**
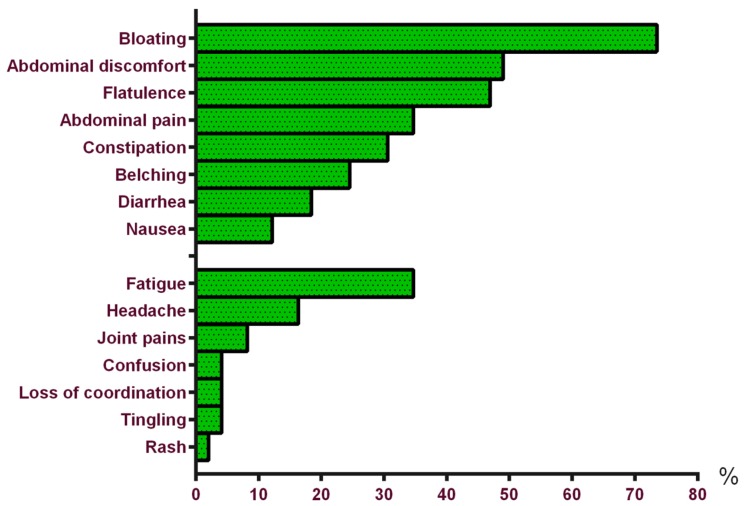
Intestinal and extra-intestinal symptoms in self-reported gluten sensitivity (*n =* 49).

The most frequently reported intestinal symptoms in srGS were bloating, abdominal discomfort and flatulence. Tiredness and headache were the most frequent extra-intestinal symptoms reported, as shown in Figure 2. Especially bread (*n =* 32, 65%), pizza (*n =* 15, 31%) and pasta (*n =* 18, 37%) induced these symptoms. Interestingly, 35% (*n =* 17) of the srGS respondents reported a reduction of clinical signs when consuming spelt bread.

**Figure 3:**
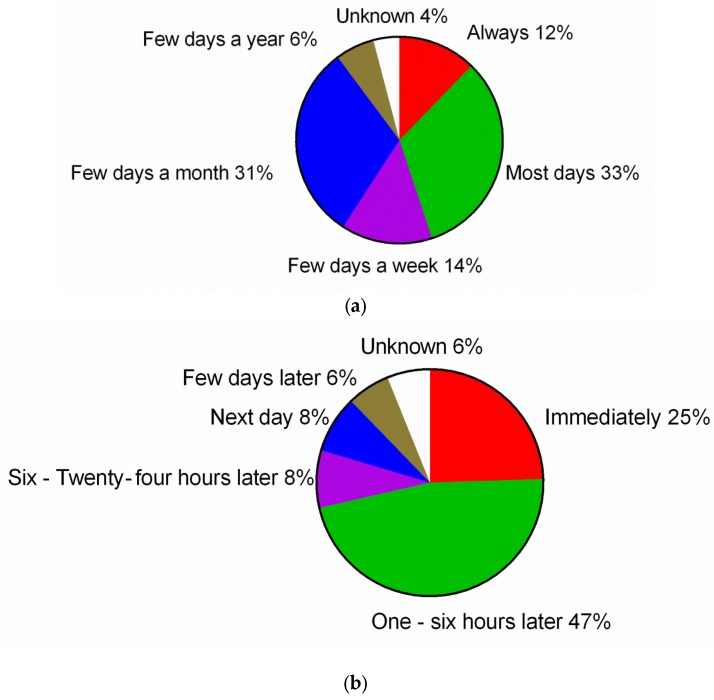

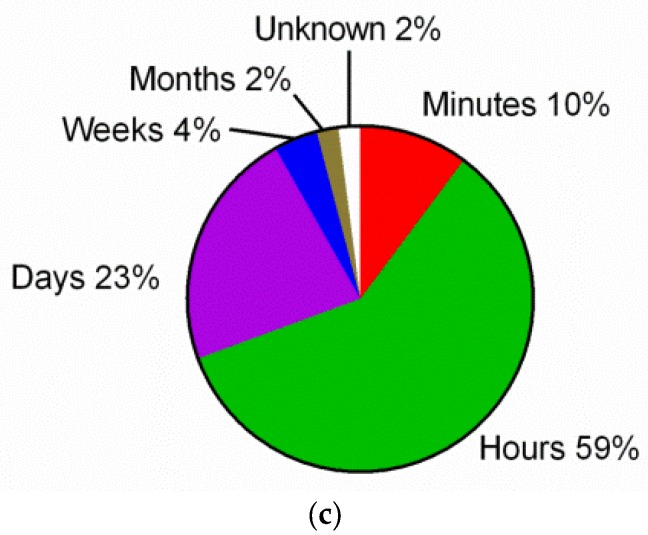
Frequency (**a**); time of onset (**b**) and duration (**c**) of srGS symptoms.

The median duration of symptoms was four years (range 0 months–40 years) at the time of the survey. Most srGS individuals (*n =* 16, 33%) reported symptoms nearly every day after eating gluten, with the onset of symptoms 1 to 6 h after ingestion of a gluten-containing food (*n =* 23, 47%). In most srGS individuals, these symptoms resolved within hours (*n =* 29, 59%). More details are shown in Figure 3.

The prevalence of individuals fulfilling the Rome III criteria for IBS in srGS was 37% vs. 9% in controls (*p <* 0.001). Medical history showed more often anxiety, anemia, chronic headache, IBS and gastro-intestinal reflux disease in srGS individuals. Table 1 shows basic demographic information and medical history. Family medical history was more often positive for CD, thyroid disease and IBS in srGS (Table 2). 

Some srGS individuals reported self-initiated dietary changes, of whom two (4%) followed a strict gluten-free diet and 21 (43%) a gluten-restricted diet. 

Eight srGS individuals (16%) visited their general practitioner, five (10%) visited a medical specialist, three (6%) visited an alternative healthcare professional, and two (4%) a dietician. The median time before consulting a healthcare professional after onset of the symptoms was two years (range 0 months–32 years). Six individuals (12%) underwent upper endoscopy examination. None of them reported a diagnosis of CD or other diagnosis after upper endoscopy examination. 

### 3.2. Fermentable Oligosaccharides, Disaccharides, Monosaccharides and Polyols (FODMAPs)

Of all srGS individuals, 74% reported abdominal discomfort related to at least one high FODMAP-containing product compared to 22% of the controls (OR 10.0 (95% confidence interval 5.2–19.3), *p <* 0.001) with a predominance of legume, cabbage, onions and leek (Table 3). 

## 4. Discussion

This study confirms that a significant part of the general adult population reports sensitivity to gluten-containing foods. The percentage is substantially less (6.2%) than in a recent comparable UK study (13%) [12]. Second, we showed that self-reported gluten sensitivity (srGS) individuals more frequently reported symptoms upon consumption of products high in FODMAPs. Third, quite surprisingly, we found that only a small number of the srGS individuals visited a doctor or ever consumed a self-initiated strict gluten-free diet (GFD).

Why srGS was found to be less prevalent in The Netherlands compared to the UK is unknown. It may be related to differences of media attention, but data to support this are lacking. 

Despite the large growth of the gluten-free market [6] and the popularity of gluten-free products, knowledge about gluten is still low in the general population as described in Australian and UK literature [17,18]. Indeed, in our survey, a high percentage of srGS individuals (35%) reported no symptoms when consuming spelt bread.

In this study, type and onset of symptoms after consumption of gluten were comparable with other non-celiac wheat sensitivity (NCWS) studies [12,15,19,20]. As shown in Table 1, anxiety, chronic headache and gastro-intestinal reflux disease were more common in the srGS group compared with controls. These subjective health complaints are also common symptoms in patients with irritable bowel syndrome (IBS) and self-reported food intolerance in general [21,22,23]. 

A significant number of NCWS patients fulfills the Rome III IBS criteria with a strong overlap between NCWS and IBS symptoms [12]. Foods which are reported to be associated with IBS symptoms are commonly rich in gluten, wheat and carbohydrates [23]. Therefore NCWS could be seen as part of IBS with a gluten-free diet as treatment strategy. Although the pathophysiology of IBS is still not well understood, food could affect a variety of physiologic parameters important in IBS such as visceral perception, motility, permeability, microbiota composition, brain-gut interactions, neuro-endocrine function and immune activation [24]. 

It is well known that stress and anxiety may exacerbate or contribute to gastrointestinal symptoms [25]. Indeed, individuals with srGS often reported an association between increase of abdominal symptoms and stress (84% vs. 48% in the control group, OR 5.7, *p <* 0.001).

The mechanisms by which gluten causes symptoms in individuals without celiac disease (CD) is unknown. There are some indications that NCWS belongs to the group of the gluten-related disorders. The relatively large number of relatives with CD in srGS individuals could indicate a shared (genetic) predisposition, although current literature is not consistent about the relationship between HLA-DQ2 and NCWS [26,27,28]. Another indication for (mild) immune activation in NCWS comes from a study which showed that serum levels of soluble CD14 and lipopolysaccharide-binding protein as well as antibody reactivity to microbial antigens are elevated in NCWS patients with resolution after a GFD [29]. 

Whether or not such immune activation is triggered by gluten is not yet established. At this point, it cannot be excluded that other ingredients in grains, including wheat germ agglutinin [30] and amylase-trypsin inhibitors [31], are in fact responsible for these signs of immune activation.

An alternative explanation is that symptoms are not immune mediated, but caused by the result of other mechanisms. One such mechanism is luminal distention of the intestine via a combination of osmotic effects and gas production caused by bacterial fermentation of poorly absorbed short-chain carbohydrates referred to as FODMAPs [32,33]. FODMAPs can be found in a variety of products, including grains. In support of the FODMAP theory, we found that individuals reporting gluten sensitivity more often reported symptoms after consuming products high in FODMAPs and less symptoms after eating spelt bread. 

It is remarkable that only a fraction of srGS individuals started a GFD and that only 16% of them had visited their general practitioner due to srGS. This indicates that apparently burden of symptoms was not severe enough to change the diet or might be related to costs and availability of gluten-free products [34]. 

## 5. Conclusions

This study confirms that gluten sensitivity is common, especially in younger individuals, females and in urban regions, although the prevalence was lower than in a comparable UK study. The high number of patients reporting symptoms in relation to FODMAPs suggests that FODMAPs are in fact responsible for part of the symptoms.

## Figures and Tables

**Table 1:** Comparison between self-reported gluten sensitivity individuals and controls: basic demographic information and medical history.

Variables	srGS (*n =* 49) (6.2%)	Controls (*n =* 734) (93.5%)	Odds Ratio (95% CI)	*p*-Value
Mean age ± sd (years)	39 ± 15.1	47 ± 18.3	-	0.001
Sex (% female)	79.6	58.3	2.8 (1.4–5.7)	<0.01
Education level (% high educated)	49.0	39.3	1.5 (0.8–2.7)	NS
Region (% urban)	75.5	55.7	2.5 (1.3–4.8)	<0.01
Rome III criteria for IBS (%)	36.7	9.0	5.9 (3.1–11.1)	<0.001
Anxiety (%)	16.3	3.1	6.0 (2.5–14.3)	<0.001
Depression (%)	14.3	8.3	1.8 (0.8–4.3)	NS
Bipolar disorder (%)	2.0	0.4	5.1 (0.5–49.7)	NS
Schizophrenia (%)	0	0.1	-	NS
Thyroid disease (%)	4.1	4.6	0.9 (0.2–3.8)	NS
Diabetes mellitus (young age onset) (%)	4.1	1.1	3.9 (0.8–18.7)	NS
Anemia (%)	16.3	6.1	3.0 (1.3–6.8)	0.01
Chronic headache (%)	6.1	3.1	2.0 (0.6–7.0)	NS
Fibromyalgia (%)	0	1.2	-	NS
Chronic fatigue syndrome (%)	2.0	0.8	2.5 (0.3–21.4)	NS
Rheumatoid arthritis (%)	4.1	4.0	1.0 (0.2–4.5)	NS
Chronic headache (%)	12.2	3.3	4.1 (1.6–10.6)	<0.01
Nut allergy (%)	2.0	1.8	1.2 (0.1–9.0)	NS
Egg allergy (%)	0	0.5	-	NS
Lactose intolerance (%)	2.0	1.4	1.5 (0.2–12.0)	NS
Inflammatory bowel disease (%)	4.1	1.2	3.4 (0.7–16.3)	NS
Gastro-intestinal reflux disease (%)	18.4	7.8	2.7 (1.2–5.8)	<0.05
Psoriasis (%)	4.1	2.0	2.0 (0.5–9.2)	NS

**Table 2:** Medical history of relatives.

Variables	srGS (*n =* 49) (6.2%)	Controls (*n =* 734) (93.5%)	Odds Ratio (95% CI)	*p*-Value
Celiac disease in all relatives (%)	8.2	2.5	3.4 (1.1–10.6)	<0.05
Celiac disease in children of srGS individuals (%)	6.1	0.4	15.9 (3.1–80.3)	<0.01
Rheumatoid arthritis (%)	30.6	20.2	1.7 (0.9–3.3)	NS
Diabetes mellitus (young age onset) (%)	14.3	7.6	2.0 (0.9–4.7)	NS
Thyroid disease (%)	20.4	8.9	2.6 (1.3–5.5)	<0.05
Psoriasis (%)	8.2	7.1	1.2 (0.4–3.4)	NS
Inflammatory bowel disease (%)	6.1	2.2	2.9 (0.8–10.4)	NS
Irritable bowel syndrome (%)	26.5	6.7	5.0 (2.5–10.1)	<0.001

**Table 3:** Comparison between self-reported gluten sensitivity individuals and controls: abdominal discomfort related to FODMAPs.

Variables	Self-Reported Gluten Sensitivity (*n =* 49) (6.2%)	Controls (*n =* 734) (93.5%)	Odds Ratio (95% Confidence Interval)	*p*-Value
Legume (%)	24.5	2.7	11.6 (5.3–25.5)	<0.001
Cabbage (%)	36.7	7.2	7.5 (3.9–14.2)	<0.001
Onion (%)	38.8	10.2	5.6 (3.0–10.4)	<0.001
Leek (%)	32.7	5.3	8.6 (4.4–17.0)	<0.001
Cauliflower (%)	22.4	3.4	8.2 (3.8–17.9)	<0.001
Mushroom (%)	12.2	2.3	5.9 (2.2–15.7)	<0.01
Apple (%)	10.2	2.0	5.4 (1.9–15.7)	<0.01
Cherry (%)	2.0	0.5	3.8 (0.4–34.7)	NS
Sugar-free gum (%)	12.2	2.7	5.0 (1.9–13.0)	<0.01
Plum (%)	10.2	3.3	3.4 (1.2–9.2)	<0.05
Pear (%)	8.2	1.8	4.9 (1.5–15.7)	<0.05
Mango (%)	2.0	0.4	5.1 (0.5–49.7)	NS
Watermelon (%)	4.1	0.3	15.6 (2.1–113.0)	<0.05
Milk (%)	20.4	4.6	5.3 (2.4–11.5)	<0.001
Buttermilk (%)	8.2	2.0	4.3 (1.4–13.4)	<0.05
Yogurt (%)	14.3	3.3	4.9 (2.0–12.1)	<0.01
Custard (%)	18.4	1.8	12.5 (5.0–30.9)	<0.001

## Supplementary Materials

The following are available online at http://www.mdpi.com/2072-6643/8/11/714/s1.
