# Supplementary material for: Prevalence and Characterization of Self-Reported Gluten Sensitivity in The Netherlands
Source: Nutrients. 2016 Nov 8;8(11):714. doi: 10.3390/nu8110714 (PMC5133100; doi:10.3390/nu8110714)
Supplement: Supplementary file 1 [file nutrients-08-00714-s001.docx]

Supplementary Materials: Prevalence and Characterization of Self-Reported Gluten Sensitivity in The Netherlands

**Tom van Gils, Petula Nijeboer, Catharina E. IJssennagger, David S. Sanders, Chris J. J. Mulder and Gerd Bouma**

Dear Sir/Madam,

**Site**

We would be grateful if you would kindly complete this 5 min questionnaire survey as part of a research project being undertaken by the Gastroenterology and Hepatology department at the VU University Medical Center, Amsterdam. This questionnaire asks about your bowel symptoms and past medical history. It is **completely anonymous** and the results are confidential and will be used only for VU University Medical Center research purposes. **There is no commercial interest**.

You may find parts of this questionnaire repetitive but please try and answer all the appropriate questions tailored for you. Should you have any queries or difficulties completing this survey, please ask our help.

Thank you for your co-operation and time in completing this questionnaire

**Part 1–This asks for basic information about yourself, any abdominal symptoms and your general state of health**

Gender: Male Female

Age: ________ years

Highest completed education level:

Elementary school Higher professional education

High school University education

Intermediate vocational education

(Q 1) Have you suffered with episodes of abdominal pains or discomfort for the
last 6 months?

Yes (Please go to Q2)

No (Please go to Q10)

(Q 2) How many days in a month do you approximately experience these
abdominal complaints?

1 day a month 4 days a month

2 days a month 5–10 days a month

3 days a month more than 10 days a month

(Q 3) Do you suffer from abdominal bloating (feeling full of gas) during the
last 6 months?

Yes No

(Q4) Do you feel an improvement in your abdominal pains or discomfort after you have emptied your bowels?

Always Often Sometimes Never

(Q5) Was the start of your abdominal pains or discomfort associated with a change in your bowel frequency?

Frequency increased Frequency decreased

No changes

(Q 6) Was the start of your abdominal pains or discomfort associated with a change in stool consistency?

Yes No

(Q 7) What was the consistency of your stool before and after the start of your abdominal complaints? (Please choose one option before and one option after).

| **Bristol Stool Chart** | | | | | | |  |
| --- | --- | --- | --- | --- | --- | --- | --- |
| **Before**  **After** | Before  After | Before  After | Before  After | Before  After | Before  After | Before  After | Before  After |
| 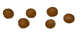 | 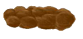 | 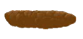 | 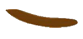 | 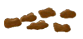 | 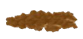 | 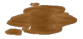 |  |
| **Separate hard lumps, like nuts (hard to pass)** | Sausage-shaped but lympy | Like a sausage but with cracks on the surface | Like a sausage or snake, smooth and soft | Soft blobs with clear-cut edges (easy to pass) | Fluffy pieces with ragged edges, a mushy stool | Watery, no solid pieces. Entirely liquid | Alternating diarrhea and constipation (hard to pass). |

(Q 8) Do your abdominal and bowel symptoms get worse with stress?

Yes No

(Q 9) Do your abdominal symptoms get worse during your menstruation?

Yes No Not applicable

(Q 10) Are you known to suffer from any of the following? (tick as many that apply)

Anxiety Chronic headache

Depression Nut allergy

Bipolar disorder Egg allergy

Schizophrenia Lactose intolerance

Thyroid disease Colon cancer

Young onset diabetes Stomach cancer

Anaemia Irritable Bowel Syndrome

Chronic fatigue Celiac disease

Fibromyalgia Crohn’s disease or Ulcerative Colitis

Chronic fatigue syndrome Gastro-intestinal reflux disease

Rheumatoid arthritis Psoriasis

**None**

**Part 2–Gluten related symptoms**

This part of the questionnaire focuses on whether you develop problems when you eat gluten. Gluten is a product found in wheat, barley or rye. Therefore, it is found in common everyday diets such as cereal, bread, cakes, biscuits, pasta, pizza etc. A small amount of the population has gluten intolerance with injury of the small bowel. This is called celiac disease. Another part of the population reports sensitivity to gluten with symptoms in the absence of small bowel injury.

(Q 11) Before starting this questionnaire, did you already know what gluten is?

Yes No

(Q 12) Before starting this questionnaire, did you already know of celiac disease?

Yes No

(Q 13) Is one of your relatives diagnosed with celiac disease?

Yes *(Please go to Q14)* No *(Please go to Q15)*

(Q 14) Which of your relatives is diagnosed with celiac disease? (tick as many that apply)

Father and/or mother

Brother(s) and/or sister(s)

One or more of your children

Grandfather and/or grandmother

Uncle, aunt and/or cousin

**None of these**

(Q 15) Is one of your relatives diagnosed with (tick as many that apply)

Rheumatoid arthritis

Young onset diabetes

Thyroid disease

Psoriasis

Crohn’s disease or Ulcerative Colitis

Irritable Bowel Syndrome

**None of these**

(Q 16a) Do you experience any symptoms which you relate to eating gluten based products?

Yes *(Please go to Q16b)* No *(Please go to* ***Q32****: Final question)*

(Q 16b) Which symptoms do you relate to eating gluten based products? (tick as many that apply)

Bloating Headaches

Abdominal pain Mental confusion

Abdominal discomfort Lack of coordination

Diarrhea Tingling

Constipation Fatigue

Belching Rash

Flatulence Joint pains

Nausea

Others (please specify):______________________________________

(Q 17) If yes, how often do you experience symptoms after eating gluten products?

Every time I eat gluten products Few times a month

On most days Few times a year

Few days a week

(Q 18) How soon after eating gluten products do you develop symptoms?

Almost immediately (less than one hour) The next day

1–6 h later Few times a year

6–24 h later

(Q 19) How long do your symptoms generally last for?

Minutes Weeks

Hours Months

Days

(Q 20) Which gluten product(s) seems to cause problems? (tick as many that apply)

Bread Pizza Cakes

Cereal Pasta Biscuits

Porridge Others (please state): ______________________

(Q 21) How long have you had a problem related to gluten? (state approximate number)

______ months OR _______ years

(Q 22) Did you have changed your diet due to your symptoms yourself without the recommendation of a dietician or medical doctor? If yes, why? If no, why?

Yes, gluten free diet

Yes, gluten restricted diet

Yes, but other change(s) than a gluten free diet

No

Because_____________________________________

(Q 23) Have you ever seen a healthcare professional due to problems related to gluten?

Yes *(Please go to Q24)* No *(Please go to Q28)*

(*Q* 24) If yes, please state whom you have seen? (tick as many that apply)

GP Medical specialist

Dietician Alternative health care professional

Other (please state) ­­­_____________________________________

(Q 25) How long did you have symptoms before visiting a healthcare professional?

______ months OR _______ years

(Q 26) Have you undergone any of the following tests to look for a cause?

Celiac blood test Yes No Not sure

Upper endoscopy Yes No Not sure

You have had no tests at all Yes

Other tests (please state) _________________________________________

(Q 27) If you’ve undergone one of the Q26 tests, have you been given any of the following results:

Celiac disease Celiac disease is excluded

Other __________________________________________

(Q 28) Have you ever tried a gluten free diet?

Yes *(Please go to Q29)* No *(Please go to Q31)*

(Q 29) If yes, was it beneficial for your symptoms?

Yes No Not sure

(Q 30) Are you still on a gluten free diet?

Yes No

(Q 31) Are there some types of bread or other cereal products reducing your symptoms? (tick as many that apply)

Spelt bread Porridge

Sourdough bread Bread containing other

grains than wheat such as barley, oats, quinoa

Other_____________________________________

There are not certain types of bread nor other cereal products that reduce
my symptoms.

(Q 32) Do you experience abdominal discomfort which you relate to eating one of these products?

Legume Plum

Cabbage Pear

Onion Mango

Leek Watermelon

Cauliflower Milk

Mushroom Buttermilk

Apple Yogurt

Cherry Custard

Sugar-free gum Other_______

**I don’t experience discomfort after eating any products.**

**You finished the questionnaire. Thank you for completing this questionnaire.**
